# Health and Economic Benefits of Air Pollution Reductions in Vietnam During 2020–2021

**DOI:** 10.3389/ijph.2023.1606238

**Published:** 2023-10-10

**Authors:** Le Tu Hoang, Vu Tri Duc, Vo Van Duc Ngoc, Ngo Xuan Truong, Nguyen Thi Nhat Thanh, Phan Thi Thu Trang, Sumeet Saksena, Nguyen Thi Trang Nhung

**Affiliations:** ^1^ Department of Biostatistics, Hanoi University of Public Health, Hanoi, Vietnam; ^2^ National Children’s Hospital, Hanoi, Vietnam; ^3^ Faculty of Information Technology, University of Engineering and Technology—Vietnam National University, Hanoi, Vietnam; ^4^ Department of Environmental Health, Hanoi University of Public Health, Hanoi, Vietnam; ^5^ East-West Center, Honolulu, HI, United States

**Keywords:** health impact assessment, economic benefits, air pollution, air quality, Vietnam

## Abstract

**Objectives:** This paper explores the potential reduction in the number of deaths and the corresponding economic benefits in Vietnam that could have arisen from the decreased in concentrations of particulate matter with a diameter of 2.5 μm or less (PM_2.5_) and nitrogen dioxide (NO_2_).

**Methods:** Using Global Exposure Mortality Models, we estimated the potential health and economic benefits on people aged 25 and above across Vietnam’s 63 provinces. The counterfactual scenario assumed reducing PM_2.5_ and NO_2_ concentrations to levels observed during the two COVID-19 epidemic waves in 2021 with national lockdowns and activity restrictions.

**Results:** In 2019, PM_2.5_ concentrations ranged from 12.8 to 40.8 μg/m^3^ while NO_2_ concentrations ranged between 2.9 and 36.98 μg/m^3^. The reduced levels of PM_2.5_ and NO_2_ resulted in 3,807 (95% CI: 2,845–4,730) and 2,451 (95% CI: 2,845–4,730) avoided deaths of adults aged 25 and above due to non-injury-related causes, respectively. Considering that every prevented death represents potential tangible and intangible cost savings, reduced levels of PM_2.5_ and NO_2_ concentrations during COVID-19 restrictions would have resulted in economic benefits of $793.0 million (95% CI: 592.7–985.4) and $510.6 million (95% CI: 381.3–634.9), respectively.

**Conclusion:** The COVID-19 lockdown led to decreased PM_2.5_ and NO_2_ concentrations, benefiting health and economy in Vietnam. Our findings highlight the potential advantages of implementing air quality control policies in the country.

## Introduction

Air pollution (AP) is a threat to health across the world, as evident in many epidemiological studies [1, 2]. Accordingly, air pollution is both the cause and the exacerbating factor of respiratory and cardiovascular diseases, cancer, stroke, and even millions of premature deaths each year globally [3, 4]. According to World Health Organization (WHO), AP is responsible for: 43% of deaths and morbidity from chronic obstructive pulmonary disease (COPD); 25% of deaths from coronary heart disease; 24% of deaths due to stroke; 17% of deaths and morbidity due to acute lower respiratory tract infections; and 29% of lung cancer deaths and illnesses [4]. In Vietnam, AP ranks in the top five risk factors for the burden of disease and premature death in 2019, just behind high blood pressure, smoking, diabetes, and nutritional risk [5].

Typically, human activities such as traffic, industrial activities and electricity generation contribute to ambient air pollution [6]. PM_2.5_ (particulate matter with aerodynamic diameter below 2.5 µm) and NO_2_ (nitro dioxide) rank as some of the most critical outdoor pollutants, as highlighted by the World Health Organization [4]. These sources can be modified by changing activity levels or reducing the intensity of the emissions [7]. In the United States, air pollution regulation has historically focused on reducing emission factors rather than modifying activity levels. While past studies have looked at how sudden changes in emission sources impact air quality [8–11], physical distancing measures introduced in response to COVID-19 provide a unique opportunity to observe and measure the effects of modifiable factors—such as large reductions in activity—on ambient air pollution in real time, with unprecedented scope, speed, and duration [12]. After the first COVID-19 case was detected, physical distancing had been implemented in Vietnam from a very early phase as one of the effective ways to mitigate the spread of COVID-19 [13]. During the physical distancing periods, Vietnam government had applied many proper solutions including residential area quarantine; closing of schools, supermarkets/markets, and restaurants; travel restrictions, public gathering restrictions, etc. [14, 15].

These solutions, which were implemented to prevent the spread of COVID-19, also reduced the emissions into the atmosphere, resulting in significantly cleaner air and, presumably, a healthier population [16–18]. A study conducted across 34 countries showed that the lockdown in 2020 reduced 31% of PM_2.5_ concentrations, leading to approximately 49,900 deaths avoided [16, 17]. Another study in 367 Chinese cities found that reductions in nitrogen dioxide (NO_2_) during the lockdown period helped avoid approximately 4,711 deaths [18]. In Vietnam, the implemented policies to control the spread of COVID-19 also improved the air quality. For instance, PM_2.5_ and NO_2_ concentrations obtained from the automatic ambient air quality monitoring station dropped by approximately 75.8% and 41.8%, respectively, during the lockdowns (1st–22nd of April 2020) in Hanoi [15]. Another study also showed that NO_2_ concentrations in Hanoi decreased between 24% and 32% in the 2 weeks after the COVID-19 lockdowns [14]. Among air pollutants, Vietnam is suffering from PM_2.5_ pollution as a result of local activities and long-range pollutant transport [15]. Despite the evidence of the air quality improvement, no study in Vietnam estimated the health effects of the change in the ambient pollution attributed to COVID-19 preventive measures.

In Vietnam, several studies have estimated the number of avoidable deaths if the air quality was controlled at certain levels [19–22]. The standard levels included the WHO air quality guidelines and the Vietnam National Ambient Air Quality Standards (denoted by QCVN 05:2013) [19–22]. On the other hand, the air pollutant reduction during COVID-19 provided an opportunity for a “natural experiment” to assess the health and economic benefits of pollution reduction. This paper aims to estimate the number of avoidable deaths and economic benefits in 63 provinces in Vietnam by reducing PM_2.5_ and NO_2_ concentrations to 2021 levels. The presumed reduction in concentration is based on the effects of lockdowns and activity restrictions.

## Methods

### Materials

#### PM_2.5_ Concentration Data

To obtain an annual average of PM_2.5_ in 2019 (before the COVID-19 pandemic) and 2021 (during COVID-19-related lockdowns and activity restrictions), the daily PM_2.5_ concentration maps in Vietnam were developed based on a Mixed Effects Model (MEM) [23] using data collected from 2012 to 2021. In 2021, there were two waves of the COVID-19 epidemic. The first wave occurred from January to April, while the second wave extended from April to December. During both waves, authorities implemented lockdowns and activity restrictions. Given the continuing occurrence of epidemic waves, we opted to select the 2021 level as the counterfactual scenario for analysis. The input data included air pollution monitoring station measurements, satellite products of Aerosol Optical Depth (AOD), meteorological maps for humidity, Planetary Boundary Layer Height (PBLH), land-use maps of traffic, normalized difference vegetation index (NDVI), and terrain.

Measured PM_2.5_ data was collected from standard stations nationwide under the management of the Northern Center of Environmental Monitoring, the US Embassy, and the Vietnam National University Ho Chi Minh City. Our satellite data consisted of AOD products of Aqua/Terra MODIS and Suomi NPP VIIRS from 2012 to 2021, obtained from the National Aeronautics and Space Administration (NASA) through their public open access sites. The meteorological data was the output of the Weather Research and Forecasting (WRF) model [24], customized for Vietnam, with the fifth generation ECMWF reanalysis (ERA-5) data used as the input. The Normalized difference vegetation index (NDVI) maps from Terra MODIS [25] were also collected from NASA from 2012 to 2021. A road map containing the shapes of road types was collected in vector format from the latest Open Street Map in 2022. Terrain map (DEM) containing information about terrain height was collected from ASTER Global source in 2019 [26].

The steps taken to develop the map included pre-processing the station measurements and maps data; enhancing the quality of the satellite AOD; integrating the map data with the station measurements; developing and validating the MEM model; estimating the daily PM_2.5_ values across the nation to make PM_2.5_ maps; aggregating the daily maps into the monthly and/or annual mean maps of PM_2.5_ for 2019 and 2021; and evaluating the maps with station measurements and comparing the outputs with the global PM_2.5_ products. Detailed methodology is presented by [27].

#### NO_2_ Concentration Data

This study used data on NO_2_ concentrations from results of Truong et al. in 2021 [28]. This data was created from validating NO_2_ satellite images of the troposphere and ground-level NO_2_ observations in Vietnam. The satellite data was collected spanning January to May of 2019 and the entirety of 2020 using the Level 2 TROPOMI sensor of the Sentinel-5P satellite. Ground observations data were collected from three automatic monitoring stations in North Vietnam (Hanoi, Quang Ninh, Viet Tri) and three in central Vietnam (Hue, Da Nang, and Nha Trang operated by the VEA (Vietnam Environment Administration), and a monitoring station in Hanoi belonging to Hanoi EPA (Environment Protection Agency). The detailed validation methodology was described by [28].

#### Population Data

The population data (*Pop*) in this study was extracted from the “*Vietnam population and housing census in 2019*” [29]. The data includes the total number of people for each age group and district in all 63 provinces of Vietnam.

#### Mortality Estimation

The age-specific mortality rate (per 100,000 population) due to non-injury causes, denoted by MR, for each district was estimated from two measurements, including the national crude mortality rate in 2019 and the injury-related mortality rate by age group [30, 31]. The first measurement was derived from the General Statistics Office webpage in Vietnam (denoted by GSO, 2019) [29]. This webpage provided the crude mortality rate (per 1,000 population) for each province in Vietnam. The second measurement was retrieved from the Vietnam National Injury Survey in 2010 (denoted by VNIS, 2010) [31]. We used the estimated mortality rate (per 100,000 population) for 5 years-age groups.

Three assumptions were made to calculate each district’s age-specific mortality rate due to all-natural causes. First, we hypothesized that the crude mortality rate was uniform within a province, meaning that within a province each district’s crude mortality rate in 2019 was the same. Second, we assumed that the injury-related mortality rate had stayed the same since 2010 as no other more recent national data were available. Third, we applied the mortality rate of each 5 years-age group from the Vietnam National Injury survey to all districts in Vietnam, meaning that the injury mortality rate of each age group is the same for every district.

Based on these assumptions, we estimate the number of deaths for non-injury causes (by age group in each district) by the following formula:

$${\mathrm{MR}}_{\left(\mathrm{non}-\mathrm{injury}\;\mathrm{causes},\mathrm{each}\;\mathrm{district}\right)}={\mathrm{MR}}_{\left(\mathrm{GSO},2019\right)}/100–{\mathrm{MR}}_{\left(\mathrm{VNIS},2010\right)}$$

where ${\mathrm{MR}}_{\left(\mathrm{non}-\mathrm{injury}\;\mathrm{causes},\mathrm{each}\;\mathrm{district}\right)}$ represents the mortality rate (per 100,000 population) due to non-injury causes for each district, *MR*_(*GSO*, *2019*)_ is the crude mortality rate (per 1,000 population) from the General Statistics Office in 2019, and *MR*_(*VNIS*, *2010*)_ is the injury mortality rate (per 100,000 population) from the Vietnam National Injury Survey in 2010. Notice that the crude mortality rate is provided as cases per 1,000 population. Thus, we divided this rate by 100 to consistently measure cases per 100,000 population.

#### Calculation of Number of Avoidable Deaths

We hypothesized that if the same interventions that had been implemeted during the COVID-19 pandemic (2021) had been implemented during 2019, the annual concentration of PM_2.5_ and NO_2_ in 2019 would have been reduced to the corresponding concentration in 2021, and the number of non-injury mortality would have declined.

##### Concentration-Response Function for PM_2.5_

We estimated the number of avoidable deaths attributed to PM_2.5_ reduction using the Global Exposure Mortality Models for non-communicable diseases (e.g., cardiovascular diseases, chronic respiratory diseases, cancer…) and lower respiratory infections (denoted by GEMM NCD + LRI) [2]. This function was chosen because it was developed based on a study in multiple areas, where the PM_2.5_ concentration is high. This function was also validated and adjusted to used in different countries. The following formula was used:

$$HR=\exp\left(\frac{\theta \log\left(\frac{{PM2.5}_{2019}-{PM2.5}_{2021}}{\alpha }+1\right)}{1+\exp(-\frac{\left({PM2.5}_{2019}-{PM2.5}_{2021}\right)-\mu }{v}}\right)$$

where HR is the hazard ratio of death due to the change of PM_2.5_ between the year of 2019 and 2021; θ *, α*, *μ* and *ν* are the parameters for each 5 years-age group (from 25 years old); $\left({PM}_{2019}-{PM}_{2021}\right)$ represents the difference obtained by subtracting the average PM_2.5_ concentration (in *µg/m* ^*3*^) for the year 2019 from the counterfactual PM_2.5_ concentration for the year 2021.

##### Concentration-Response Function for NO_2_

For NO_2_, we applied the log-linear model with a beta coefficient ( β ), the natural logarithm of the dose-response function (the relative risk) expressed for a 10 μg/m^3^ increase in exposure of the air pollutant between the baseline and the counterfactual time points, recommended by the World Health Organization [32]. The formula was:

$$RR=e^{-\beta *\left({NO2}_{2019}-{NO2}_{2021}\right)}$$

where *RR* is the relative risk of death due to the change of *NO*_2_; *β* is the slope of the model; and $\left({NO2}_{2019}-{NO2}_{2021}\right)$ is the difference between the annual average of NO_2_ concentration (in *µg/m* ^*3*^) in 2019 and the counterfactual concentration in 2021. In this study, RR was 1.041 with 95% CI: (1.019; 1.064) as recommended for calculation with NO_2_ [32].

##### Number of Avoidable Deaths

The number of avoidable deaths attributed to the reduction of air pollutant concentrations was estimated as outlined below:

$$AN=\sum \left(\left(1-\frac{1}{HR\;\left(or\;RR\right)}\right)*{MR}_{i,j}*{Pop}_{i,j}\right)$$

where *AN* is the avoidable number of deaths for each province/city, *HR* (or *RR*) is the hazard ratio (or the relative risk) estimated from the concentration-response function, *MR*_*i*,*j*_ and *Pop*_*i*,*j*_ is the mortality rate and the total number of people of *i* age group and *j* district. This figure is reported along with its 95% confidence interval for each province, ecological zone, and region in Vietnam. The 95% confidence intervals were calculated using Monte Carlo simulation in BENMAP-CE software.

#### Economic Value Calculation

We used the value of a statistical life (VSL) approach to calculate the economic benefits of premature death. We calculated VSL using the individual willingness to pay (WTP) to decrease the risk of death [33]. This figure considers the intangible costs, including the value of suffering and leisure time lost, and demonstrates the tangible costs of medical treatment [34]. To assess the economic benefits in this study, we multiplied the predicted number of avoided deaths by a locally valid estimation of VSL [35]. Ideally, local studies should be utilized to estimate the economic loss. Owing to the absence of such local studies, however, we used the benefit-transfer approach to transfer unit health costs from foreign studies to the local context [33, 36] by using the formula:

$${\mathrm{VSL}}_{\mathrm{Vietnam},2019}={\mathrm{VSL}}_{\mathrm{OECD},2011}\times {\left(Y_{\mathrm{Vietnam},2019}/Y_{\mathrm{OECD},2019}\right)}^{b}$$

where Y is GDP (gross domestic product) *per capita*, and ***b*** is the income elasticity of VSL. For low- and middle-income countries, ***b*** differs from 1.0 to 1.4, with a central estimate of 1.2 [33]. This study used the ***b*** parameter of 1.2. In this calculation, *VSL*_*OECD*,*2011*_ equals $3,832,843 (World Bank [37], at 2011 market rates, PPP); *Y*_*Vietnam*,*2019*_ equals $3,491.1 (World Bank [38], Per capita, 2019); *Y*_*OECD*,*2019*_ equals $39,531.7 (World Bank [38], Per capita, 2019). The result for *VSL*_*Vietnam*,*2019*_ was $208,324.9 (at 2019 market rates).

The health economic value was calculated by multiplying the number of avoided deaths with an estimated economic value per case (***VSL***_***Vietnam, 2019***_)—this estimated to be approximately $208,324.9. All calculations were done using BenMAP-CE 1.5.8 provided by the US Environmental Protection Agency (US EPA) [39]. We also used QGIS 3.30 to illustrate the results in three different regions (Northern, Central, and Southern Vietnam).

#### Ethical Considerations

The studies involving human participants were reviewed and approved by The Ethics Committee of Hanoi University of Public Health under Decision No. 318/2020/YTCC-HD3 dated 30 July 2020. The ethics committee waived the requirement of written informed consent for participation.

## Results

### Comparison of PM_2.5_ and NO_2_ Concentration Before and After COVID-19 in Vietnam

**FIGURE 1:**
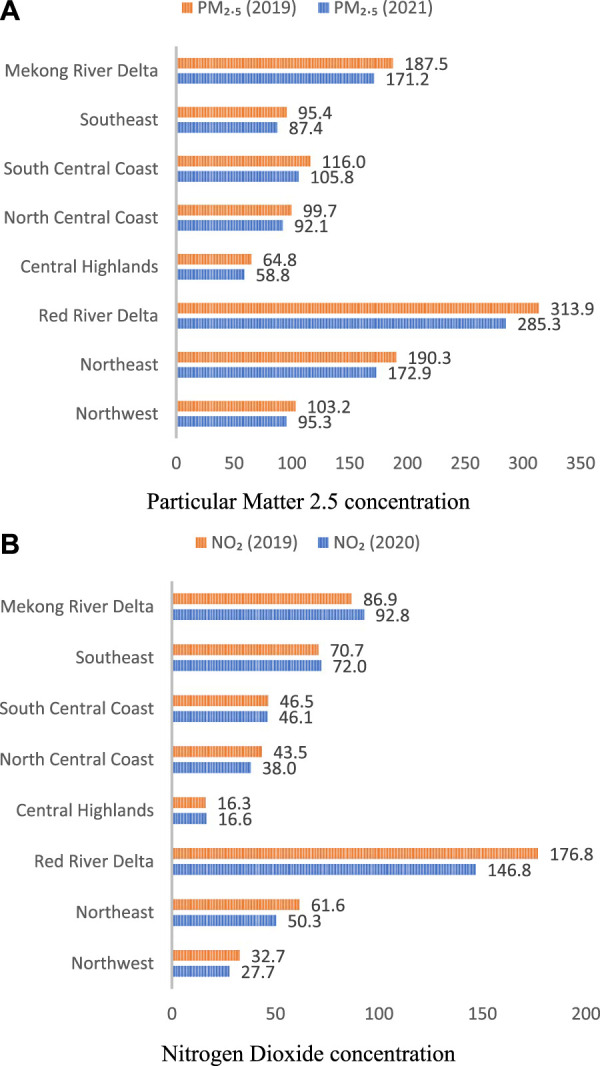
Annual average particular matter 2.5 **(A)** and nitrogen dioxide **(B)** concentrations by ecological zones (Vietnam. 2019–2021).

The results, presented in Figure 1, indicate that the Red River Delta region in the northern part of the country had the highest levels of PM_2.5_ and NO_2_ in all years. In 2019, the annual average PM2.5 concentrations in these provinces ranged from 12.8 to 40.8 μg/m^3^, whereas in 2021 they ranged from 11.5 to 36.9 μg/m^3^. The NO_2_ concentrations ranged from 2.9 to 36.9 μg/m^3^ in 2020 and from 2.8 to 36.1 μg/m^3^ in 2021, indicating some reduction in NO_2_ levels over the study period. Compared to 2019, the average PM_2.5_ concentrations in all ecological zones decreased in 2021, whereas NO_2_ concentrations decreased in only five of the eight zones, including the Red River Delta, Northeast, Northwest, North and South-Central coast. The reductions in annual PM2.5 concentrations across the 63 provinces of Vietnam ranged from 5.7% to 10.3%. Detailed information of PM_2.5_ and NO_2_ concentration for each province was described in Supplementary Table S1.

Table 1 shows that there was a reduction in PM_2.5_ concentrations in all ecological zones, ranging from 7.6% in the Northeast zone to 9.3% in the North central coast zone. In the case of NO_2_, the reductions varied across zones, ranging from 0.9% in the Central Highlands to 18.3% in the Northwest zone. However, due to agriculture activities (burning straw, crop residues and biomass), there was an increase in NO_2_ concentrations in some zones, including the North Central Coast (2.1%), Southeast (1.8%), and Mekong River Delta (6.8%).

**TABLE 1:** Change in total concentrations of air pollutants by ecological zones (Vietnam. 2019–2021).

Region	Ecological zones	Change in total concentration (%)	
		(Positive value: increase, negative value: decrease)	
		PM_2.5_ (2019 vs. 2021)	NO_2_ (2019 vs. 2020)
Northern Vietnam	Northeast	−7.6	−15.2
	Northwest	−9.1	−18.3
	Red River Delta	−9.1	−17.0
	Total	−8.9	−17.1
Central Vietnam	North central coast	−9.3	2.1
	South central coast	−7.6	−12.5
	Central highlands	−8.7	−0.9
	Total	−8.4	−5.2
Southern Vietnam	Southeast	−8.4	1.8
	Mekong River Delta	−8.7	6.8
	Total	−8.6	4.6
All		−8.7	−8.3

### Crude Mortality Rate in Vietnam in 2019

**FIGURE 2:**
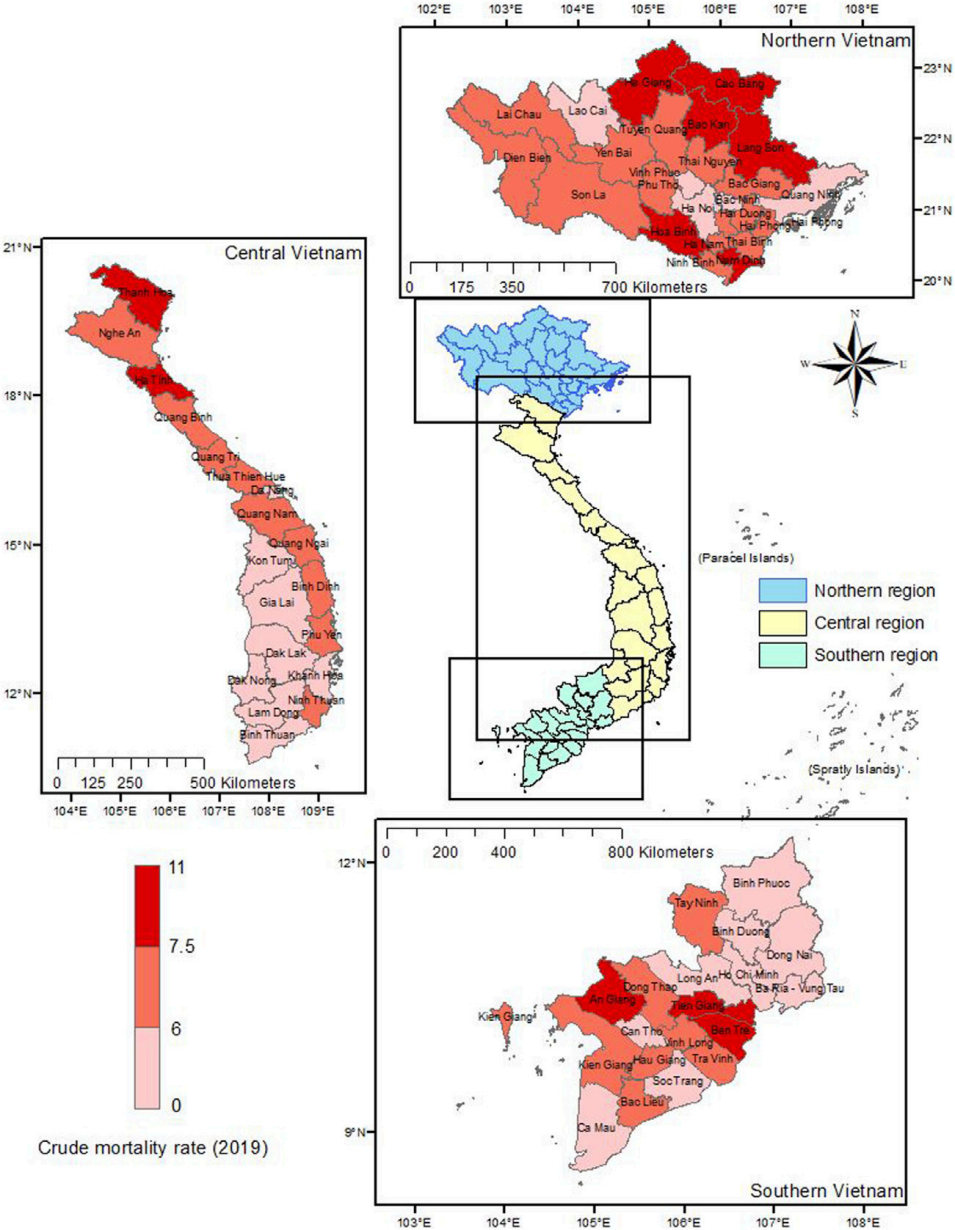
Crude mortality rate by province (Vietnam. 2019).

Figure 2 displays the crude mortality rates of each Vietnamese province in 2019. The highest crude mortality rates were observed in the northern provinces, followed by the Central region and Southern region. In contrast, the Central highlands, including provinces such as Kon Tum, Gia Lai, Dak Lak, and Dak Nong, had lower mortality rates, with approximately less than six deaths per 1,000 population.

### Avoidable Deaths

**FIGURE 3:**
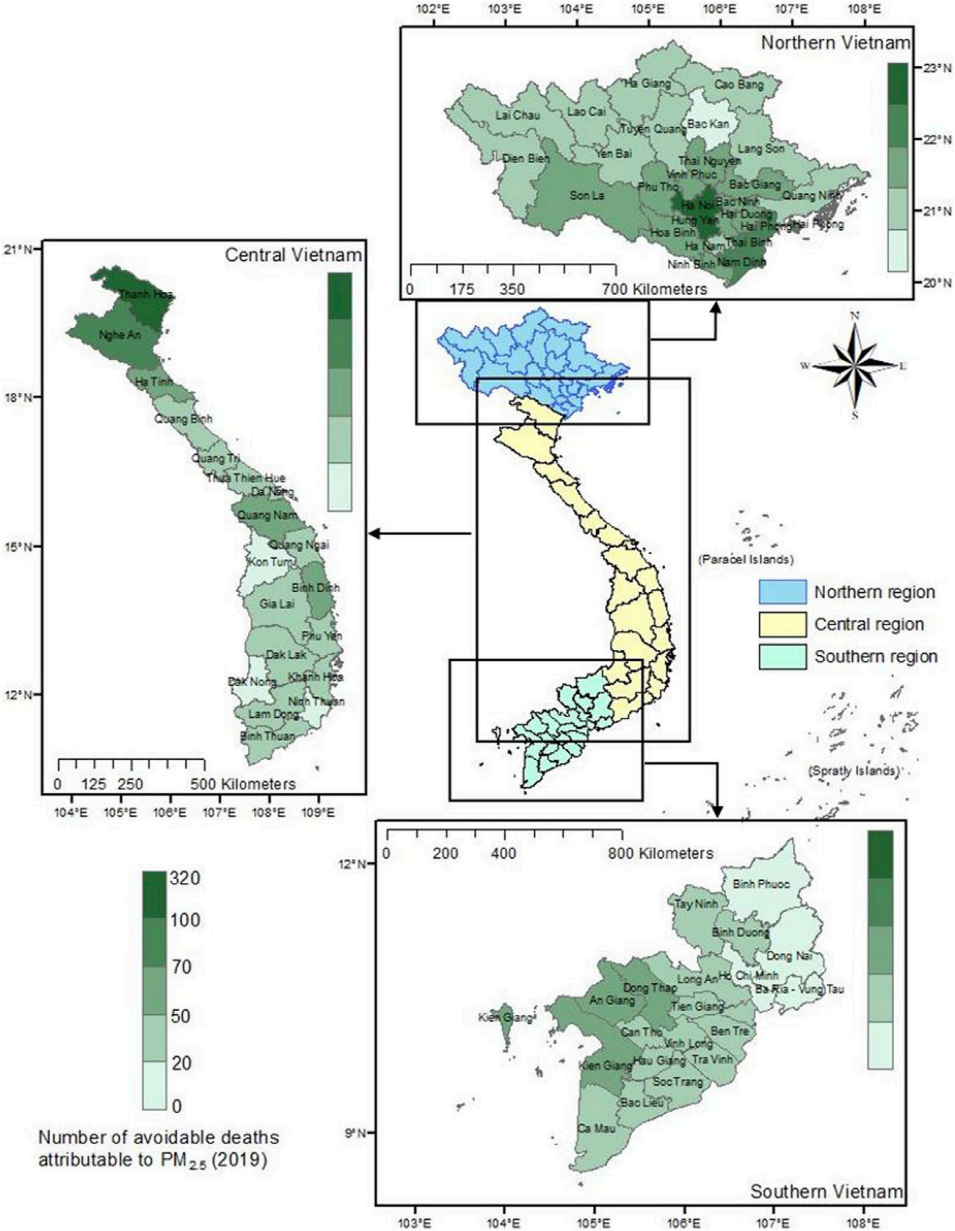
Number of avoidable deaths in 2019 by province if particular matter 2.5 concentrations were reduced to 2021 levels (Vietnam. 2019).

Figure 3 displays the number of avoidable deaths attributed to the reduction in PM_2.5_ concentrations in 2019. The highest number of avoidable deaths due to PM_2.5_ was observed in the Northern region, with a total of 1,990 deaths (95% CI: 1,488–2,472). In the Central region, this figure was lower at 1,071 deaths (95% CI: 801–1,331), while the Southern part of Vietnam had the lowest number of avoidable deaths at 745 (95% CI: 557–926). If air quality management measures had been implemented to maintain the air quality as it was during the COVID-19 pandemic, the highest number of avoidable deaths in 2019 would have been 430 cases (95% CI: 330–540). The province with the lowest avoidable deaths was Ba Ria–Vung Tau in the south, with only about 7 deaths (95% CI: 5.1–8.5). In the central areas of Vietnam, Thanh Hoa had the highest number of avoidable deaths, with approximately 250 cases (95% CI: 190–310).

### Avoidable Deaths When Adjusting the NO_2_ Concentration in Vietnam

**FIGURE 4:**
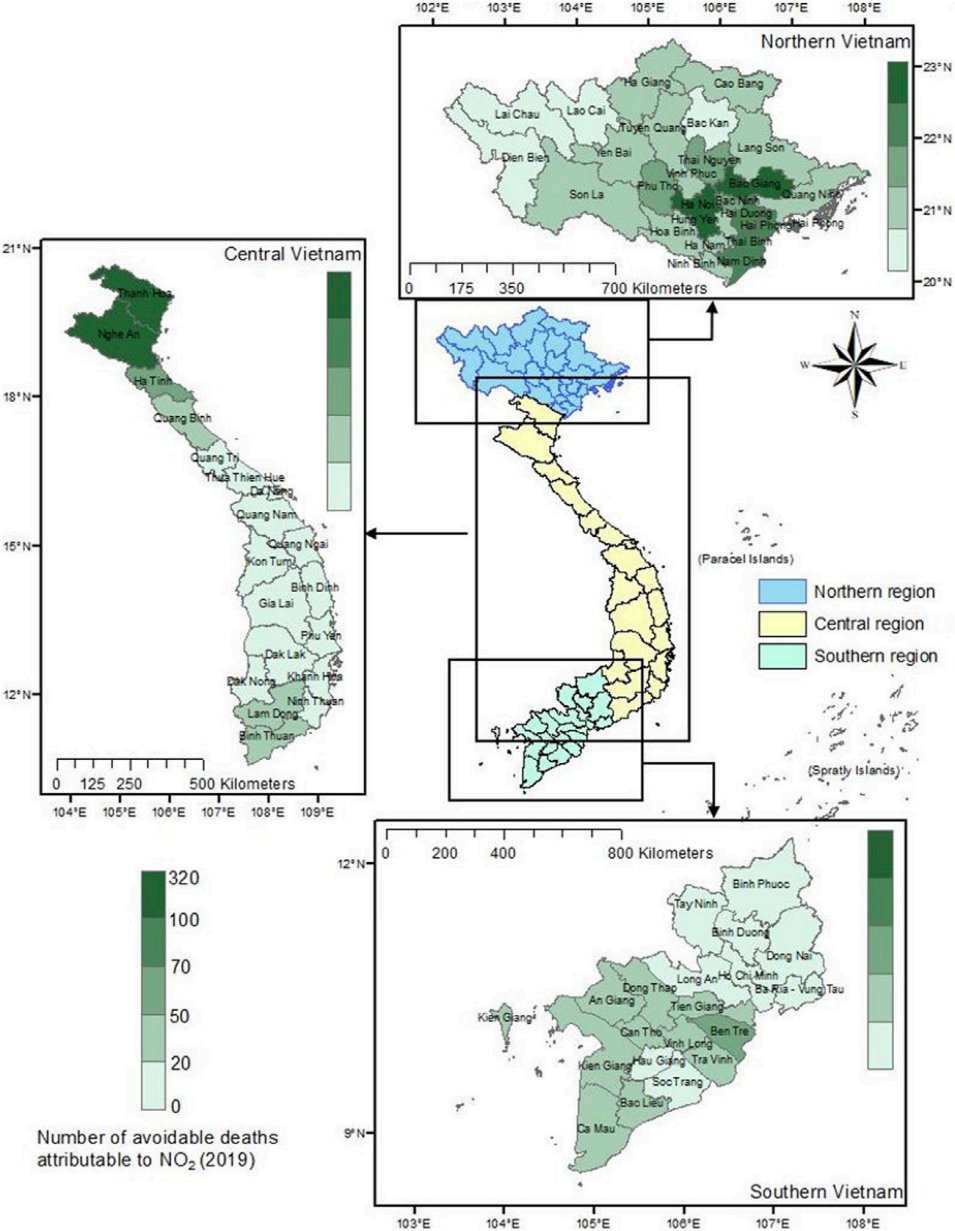
Number of avoidable deaths in 2019 by province if nitro dioxide concentrations were reduced to 2021 levels (Vietnam. 2019).

Figure 4 illustrates the total number of avoidable deaths attributed to NO_2_. Similar to the trend observed for PM2.5, the Northern region of Vietnam had the highest number of avoidable deaths, with 1,494 deaths (95% CI: 1,117–1,857), while the Southern region had the lowest, with only 421 avoidable deaths (95% CI: 314–524). The highest number of avoidable deaths was seen in Ha Noi (315 avoidable deaths, 95% CI: 236–392), Thanh Hoa (174 avoidable deaths, 95% CI: 130–217), Nghe An (130 avoidable deaths, 95% CI: 98–163), and Bac Giang (107 avoidable deaths, 95% CI: 80–133). Compared to these provinces, the mortality rates in the Southern provinces were significantly lower, ranging from 3 (Ba Ria–Vung Tau) to 37 (Tien Giang) avoidable deaths. Even in Ho Chi Minh City, only approximately 5 deaths could have been avoided.

### Economic Benefits

Table 2 displays the total number of avoidable deaths and corresponding economic benefits for each region in Vietnam. Considering that economic benefits represent the intangible costs, including the value of suffering and leisure time lost, and demonstrates the tangible costs of medical treatment. Overall, reducing PM_2.5_ concentrations could have prevented approximately 3,807 deaths (95% CI: 2,845–4,730) in Vietnam in 2019, resulting in potential savings of up to $793.0 million (95% CI: 592.7–985.4). The Red River Delta region would have benefited the most economically from this reduction, with potential savings of about $414.6 million. Additionally, reducing NO_2_ concentrations could have prevented a total of 2,451 deaths (95% CI: 1,830–3,047), resulting in potential savings of approximately $510.6 million (95% CI: 381.3–634.9). Once again, the Red River Delta region would have benefited the most economically from this reduction, with potential savings of almost $311.3 million.

**TABLE 2:** Economic benefits of air pollutants concentration reduction (Vietnam. 2019).

Region	Ecological zones	PM_2.5_		NO_2_	
		No. of avoidable deaths	Economic value ($ million)	No. of avoidable deaths	Economic value ($ million)
Northern Vietnam	Northeast	497 (372–618)	103.6 (77.4–128.7)	413 (308–513)	85.9 (64.2–106.8)
	Northwest	247 (184–307)	51.4 (38.4–63.9)	172 (128–213)	35.8 (26.7–44.4)
	Red River Delta	1,246 (932–1,548)	259.7 (194.1–322.5)	910 (680–1,131)	189.6 (141.7–235.6)
	Total	1990 (1,488–2,472)	414.6 (310.0–515.0)	1,494 (1,117–1,857)	311.3 (232.6–386.9)
Central Vietnam	North Central Coast	628 (469–780)	130.7 (97.7–162.4)	415 (310–516)	86.5 (64.6–107.5)
	South Central Coast	299 (223–371)	62.2 (46.5–77.3)	81 (60–101)	16.8 (12.5–20.9)
	Central Highlands	145 (108–180)	30.2 (22.6–37.6)	40 (30–50)	8.3 (6.2–10.3)
	Total	1,071 (801–1,331)	223.2 (166.8–277.4)	536 (400–666)	111.6 (83.4–138.8)
Southern Vietnam	Southeast	141 (106–176)	29.5 (22.0–36.6)	65 (48–80)	13.4 (10.0–16.7)
	Mekong River Delta	604 (451–751)	125.8 (94.0–156.4)	356 (265–444)	74.2 (55.3–92.4)
	Total	745 (557–926)	155.2 (116.0–193.0)	421 (314–524)	87.6 (65.3–109.2)
All		3,807 (2,845–4,730)	793.0 (592.7–985.4)	2,451 (1,830–3,047)	510.6 (381.3–634.9)

## Discussion

Our study is among the few studies to perform a nation-wide health impact assessment of air pollution in Vietnam. There have been several prior studies assessing the burden of mortality attributed to air pollution, typically PM_2.5_ [19–22]. However, these studies only estimated the burden of PM_2.5_ attributable deaths in smaller regions of the country, for example, in the megacities such as Hanoi [20] and Ho Chi Minh city [19, 22]. A recent study conducted such an assessment for multiple sites, including ten provinces in Northern Vietnam, and Ho Chi Minh City (which is in the south) [21]. However, such studies used the WHO Air Quality Guidelines (5 and 10 μg/m^3^ for annual PM_2.5_ and NO_2_, respectively) and the Vietnam National Ambient Air Quality Standards (QCVN 05:2013) (25 and 40 μg/m^3^ for annual PM_2.5_ and NO_2_, respectively) as the counterfactual level to estimate the attributable burden of air pollution [19–22]. These levels, however, were challenging to attain not only for recent periods but also in the near future since the air pollution level in 2019 of many provinces, especially in the Red River Delta, far exceeded the QCVN 05:2013 standards. While another study highlighted the failure of provinces to meet the WHO air quality guidelines [40], our results demonstrated that PM_2.5_ exceeded by 7–30 μg/m^3^ and NO_2_ by 1–26 μg/m^3^ in the majority of provinces. In contrast, we assessed the impact using the air pollutant concentration in 2021 as a counterfactual level. This level was achieved by implementing stringent interventions to prevent the spread of COVID-19. Thus, applying this realistically achievable level may provide more practical evidence of health benefits attributed to air pollution reduction.

In Vietnam, some primary emission sources of PM_2.5_ include agricultural by-product burning, traffic, industrial activities, and craft villages [41]. Craft villages in Vietnam contribute to air pollution emissions through their traditional production techniques, which involve the burning of diverse materials and fuels. Examples of such villages include pottery-making communities, incense stick crafting villages, and blacksmithing villages, all of which engage in practices that result in the release of pollutants into the atmosphere. During the COVID-19 pandemic, public health and social measures had been applied by the Vietnam government, including: work from home agreements, school closures, restriction on public gatherings, lockdowns, border closures, isolation, and quarantine areas [42]. Therefore, limiting daily activities inevitably led to reducing PM_2.5_ emissions [14–16]. For NO_2_, a recent study showed that the restriction of anthropogenic activities during the COVID-19 pandemic led to the decline of concentration, and the northern area in Vietnam observed a higher reduction in NO_2_ concentration than the south [14, 15, 28]. These results are consistent with our findings.

Air pollution reduction attributed to the constraint of human activities has helped gain health benefits, including a decrease in mortality. If Vietnam had applied stringent measures to control the PM_2.5_ and NO_2_ emissions in 2019 to reach 2021 levels, more than 3,000 and 2,000 deaths could have been avoided, respectively. The highest benefits were obtained in northern Vietnam, especially the Red River Delta region. This region is populous, with a population density reaching about 1,000 people/km^2^ [43]. It also includes Hanoi (the capital city of Vietnam) and other central provinces such as Bac Ninh, Thai Binh, Hung Yen, where anthropogenic activities happen frequently.

Moreover, we also estimated the economic benefit of air pollution reduction corresponding to the mortality decline. The result for Ho Chi Minh city was lower than what was found in other recently published research [22]. Several factors can explain this difference in findings. The first reason might be that while the previous research used air quality guidelines and QCVN 05:2013 as counterfactual level [22], our study measured the change in air pollution due to COVID-19 preventive measures, which only range from 0.47 to 2.29 μg/m^3^ for PM_2.5_. Even though this minor change in air pollution contributes to a smaller change in economic benefit, the burden estimated in this study remains significant. Another reason for the difference in results may be the use of different concentration-response functions. The prior research used the pooled effect of many other studies [22], while we used the global exposure mortality model (denoted as GEMM) [2]. Although many other approaches can be used to conduct health impact assessment [1, 33, 44], each calculation has advantages and limitations, therefore it is better to have a consistent measurement throughout the assessment in Vietnam to compare the result. Thus, national health impact assessment guidelines for each disease and each pollutant are essential.

### Limitations

Our research had several limitations. First, we assumed that the mortality rate from the Vietnam National Injury Survey had stayed the same since 2010. A study found that the trend of deaths attributed to injury in Vietnam had not been significantly different from 2007 to 2017 [45]. Thus, this approach is acceptable, although it might introduce a certain amount of uncertainty. Second, we did not account for the impact of indoor air pollution because the required data and evidence for such an estimation were not available. We acknowledge that people may have had higher exposures to pollutants at home due to quarantines and physical distancing. It is an important consideration for future studies because indoor air pollution can significantly impact human health [46]. Third, the estimation for Vietnam Value of Statistical Life is still based on a non-updated database (from 2011) and OECD-standard price, so it might not reflect the actual economic benefits when controlling the air quality in Vietnam. However, our findings suggest further studies to construct a more fitting VSL within regions sharing greater similarities with Vietnam.

### Conclusion

The implementation of COVID-19 transmission control measures such as lockdowns and social restrictions in Vietnam in 2021 led to a reduction in PM_2.5_ and NO_2_ concentration levels, particularly in provinces located in the Red River Delta region. This reduction suggests that if similar air quality levels were maintained in 2019, mortality could have been avoided, with potential economic benefits of $793.0 million (95% CI: 592.7–985.4) and $510.6 million (95% CI: 381.3–634.9) attributable to PM_2.5_ and NO_2_ concentration reductions, respectively. To better estimate health and economic benefits, there is a need for stronger public policies aimed at controlling air quality in Vietnam, as well as increased investment in automatic monitoring stations and health data collection, when calculations heavily relied on the precision of pollutant concentrations and mortality rates. Additionally, a reassessment of the Vietnam Value of Statistical Life using more recent parameters would enhance its precision.
