# Supplementary material for: Health and Economic Benefits of Air Pollution Reductions in Vietnam During 2020–2021
Source: Int J Public Health. 2023 Oct 10;68:1606238. doi: 10.3389/ijph.2023.1606238 (PMC10593963; doi:10.3389/ijph.2023.1606238)
Supplement: Supplementary file 1 [file Table1.docx]

**Health impact assessment and economic benefits of reductions in PM_2.5_ and NO_2_ concentrations in Vietnam during the COVID-19 pandemic**

Table S1. Annual PM_2.5_ and NO_2_ concentration by province in Vietnam

| Region | Ecological Zone | Province | Average PM2.5 (annual) | | Average NO_2_ (annual) | |
| --- | --- | --- | --- | --- | --- | --- |
|  |  |  | 2019 | 2021 | 2019 | 2020 |
| Northern Vietnam | Northeast | Bac Giang | 30.46 | 27.91 | 10.38 | 8.54 |
| Northern Vietnam | Northeast | Phu Tho | 30.40 | 27.37 | 11.09 | 7.98 |
| Northern Vietnam | Northeast | Thai Nguyen | 24.67 | 22.35 | 8.21 | 7.49 |
| Northern Vietnam | Northeast | Quang Ninh | 18.93 | 17.25 | 6.46 | 5.35 |
| Northern Vietnam | Northeast | Yen Bai | 19.78 | 18.28 | 7.50 | 5.07 |
| Northern Vietnam | Northeast | Cao Bang | 16.40 | 14.85 | 5.10 | 4.71 |
| Northern Vietnam | Northeast | Lang Son | 15.96 | 14.48 | 4.70 | 4.34 |
| Northern Vietnam | Northeast | Bac Kan | 16.30 | 14.65 | 4.04 | 3.56 |
| Northern Vietnam | Northeast | Ha Giang | 17.40 | 15.81 | 4.10 | 3.26 |
| Northern Vietnam | Northwest | Hoa Binh | 16.94 | 15.40 | 8.43 | 6.59 |
| Northern Vietnam | Northwest | Lao Cai | 17.96 | 16.57 | 6.81 | 4.74 |
| Northern Vietnam | Northwest | Dien Bien | 15.77 | 14.87 | 3.92 | 4.70 |
| Northern Vietnam | Northwest | Tuyen Quang | 21.05 | 19.05 | 6.41 | 4.35 |
| Northern Vietnam | Northwest | Lai Chau | 16.16 | 15.20 | 3.38 | 3.90 |
| Northern Vietnam | Northwest | Son La | 15.29 | 14.23 | 3.75 | 3.44 |
| Northern Vietnam | Red River Delta | Ha Noi | 40.80 | 36.90 | 27.23 | 23.92 |
| Northern Vietnam | Red River Delta | Ha Nam | 31.51 | 28.54 | 22.10 | 19.22 |
| Northern Vietnam | Red River Delta | Hung Yen | 35.20 | 31.81 | 20.71 | 17.87 |
| Northern Vietnam | Red River Delta | Nam Dinh | 26.45 | 24.38 | 19.39 | 15.11 |
| Northern Vietnam | Red River Delta | Ninh Binh | 23.46 | 21.17 | 18.53 | 14.33 |
| Northern Vietnam | Red River Delta | Bac Ninh | 37.83 | 34.65 | 16.40 | 14.08 |
| Northern Vietnam | Red River Delta | Hai Phong | 25.81 | 23.55 | 18.12 | 13.45 |
| Northern Vietnam | Red River Delta | Vinh Phuc | 34.55 | 31.32 | 13.15 | 12.58 |
| Northern Vietnam | Red River Delta | Thai Binh | 27.06 | 24.66 | 11.65 | 9.15 |
| Northern Vietnam | Red River Delta | Hai Duong | 31.24 | 28.28 | 9.55 | 7.06 |
| Central Vietnam | North Central Coast | Thanh Hoa | 19.16 | 17.36 | 11.54 | 8.89 |
| Central Vietnam | North Central Coast | Nghe An | 17.08 | 15.73 | 8.94 | 7.79 |
| Central Vietnam | North Central Coast | Ha Tinh | 17.58 | 16.34 | 7.51 | 6.39 |
| Central Vietnam | North Central Coast | Thua Thien Hue | 15.94 | 14.77 | 5.97 | 5.57 |
| Central Vietnam | North Central Coast | Quang Tri | 15.18 | 14.25 | 4.88 | 5.10 |
| Central Vietnam | North Central Coast | Quang Binh | 14.73 | 13.70 | 4.60 | 4.29 |
| Central Vietnam | South Central Coast | Da Nang | 18.84 | 17.38 | 14.46 | 13.32 |
| Central Vietnam | South Central Coast | Ninh Thuan | 13.57 | 12.33 | 4.72 | 5.82 |
| Central Vietnam | South Central Coast | Quang Nam | 14.92 | 13.76 | 4.91 | 4.80 |
| Central Vietnam | South Central Coast | Binh Thuan | 13.22 | 12.30 | 4.33 | 4.79 |
| Central Vietnam | South Central Coast | Binh Dinh | 13.86 | 12.57 | 4.80 | 4.69 |
| Central Vietnam | South Central Coast | Phu Yen | 13.41 | 12.04 | 4.96 | 4.52 |
| Central Vietnam | South Central Coast | Quang Ngai | 14.53 | 13.20 | 4.75 | 4.38 |
| Central Vietnam | South Central Coast | Khanh Hoa | 13.63 | 12.24 | 3.62 | 3.78 |
| Central Vietnam | Central Highlands | Dak Lak | 13.11 | 11.76 | 3.48 | 3.63 |
| Central Vietnam | Central Highlands | Gia Lai | 13.20 | 12.01 | 3.37 | 3.55 |
| Central Vietnam | Central Highlands | Dak Nong | 12.75 | 11.55 | 3.22 | 3.38 |
| Central Vietnam | Central Highlands | Lam Dong | 12.77 | 11.56 | 3.34 | 3.27 |
| Central Vietnam | Central Highlands | Kon Tum | 12.98 | 11.93 | 2.86 | 2.79 |
| Southern Vietnam | Southeast | Ho Chi Minh | 20.87 | 18.91 | 36.93 | 36.12 |
| Southern Vietnam | Southeast | Binh Duong | 15.98 | 14.55 | 10.92 | 11.44 |
| Southern Vietnam | Southeast | Dong Nai | 14.13 | 12.97 | 6.67 | 7.31 |
| Southern Vietnam | Southeast | Ba Ria - Vung Tau | 14.00 | 13.00 | 6.77 | 7.07 |
| Southern Vietnam | Southeast | Tay Ninh | 16.82 | 15.44 | 5.80 | 6.15 |
| Southern Vietnam | Southeast | Binh Phuoc | 13.60 | 12.54 | 3.66 | 3.96 |
| Southern Vietnam | Mekong River Delta | Can Tho | 14.92 | 13.62 | 10.49 | 11.02 |
| Southern Vietnam | Mekong River Delta | Vinh Long | 14.84 | 13.74 | 8.56 | 9.48 |
| Southern Vietnam | Mekong River Delta | Tien Giang | 15.29 | 13.99 | 8.24 | 8.84 |
| Southern Vietnam | Mekong River Delta | Dong Thap | 15.53 | 14.24 | 8.18 | 8.61 |
| Southern Vietnam | Mekong River Delta | Ben Tre | 14.28 | 13.14 | 6.63 | 7.51 |
| Southern Vietnam | Mekong River Delta | An Giang | 14.75 | 13.51 | 7.48 | 7.48 |
| Southern Vietnam | Mekong River Delta | Long An | 15.80 | 14.40 | 6.70 | 7.29 |
| Southern Vietnam | Mekong River Delta | Bac Lieu | 13.89 | 12.59 | 5.84 | 6.51 |
| Southern Vietnam | Mekong River Delta | Soc Trang | 13.49 | 12.16 | 5.55 | 5.96 |
| Southern Vietnam | Mekong River Delta | Hau Giang | 13.93 | 12.56 | 5.53 | 5.69 |
| Southern Vietnam | Mekong River Delta | Tra Vinh | 13.68 | 12.58 | 5.15 | 5.29 |
| Southern Vietnam | Mekong River Delta | Kien Giang | 13.60 | 12.39 | 4.80 | 5.04 |
| Southern Vietnam | Mekong River Delta | Ca Mau | 13.52 | 12.25 | 3.71 | 4.07 |
